# Effects of isoquinoline alkaloids from *Macleaya cordata* on growth performance, survival, immune response, and resistance to *Vibrio parahaemolyticus* infection of Pacific white shrimp (*Litopenaeus vannamei*)

**DOI:** 10.1371/journal.pone.0251343

**Published:** 2021-05-06

**Authors:** Pavarist Bussabong, Tirawat Rairat, Niti Chuchird, Arunothai Keetanon, Putsucha Phansawat, Kanokwan Cherdkeattipol, Phongchate Pichitkul, Waraporn Kraitavin

**Affiliations:** 1 Faculty of Fisheries, Department of Fishery Biology, Kasetsart University, Bangkok, Thailand; 2 Faculty of Fisheries, Department of Aquaculture, Kasetsart University, Bangkok, Thailand; 3 Phytobiotics (Thailand) Co., Ltd, Huaykwang, Bangkok, Thailand; Kafrelsheikh University, EGYPT

## Abstract

Isoquinoline alkaloids (IQs) from *Macleaya cordata* are promising natural products for enhancing the growth performance and overall health condition of farmed animals. The present study aimed to investigate the effects of two formulas of IQ extract, provided in either a powdered formula (IQ-E) or a water-soluble, granulated formula (IQ-WS) and containing the main active component sanguinarine at a concentration of 0.5% and 1%, respectively, on the growth, survival, immune response, and resistance to *Vibrio parahaemolyticus* infection of Pacific white shrimp (*Litopenaeus vannamei*). In Experiment 1, the postlarvae were divided into five groups (four replicates/group and 100 shrimp/tank) and fed four times/day for 30 days with a control feed, IQ-E at 200 or 300 mg/kg of feed, or IQ-WS at 100 or 150 mg/kg of feed. In Experiment 2, the surviving shrimp from Experiment 1 were redistributed into six groups (four treatment groups as in Experiment 1 plus the positive and negative controls with four replicates/group and 30 shrimp/tank) and challenged with *V*. *parahaemolyticus* by immersion at a concentration of 10^3^ colony-forming units (CFU)/mL and were fed with the same diets for another 14 days. The results revealed that all IQ-fed shrimp in Experiment 1 had significantly enhanced survival rates and immune parameters (total hemocyte count and phagocytic, phenoloxidase, and superoxide dismutase activities) compared to the control group, even though the growth performances were similar across all groups. In Experiment 2, all IQ-fed groups showed better growth performance and survival rates compared to the positive control. Other than in the positive control group, no histopathological lesions in the hepatopancreas and the intestine were found. In summary, the current study demonstrated the benefits of using IQs from *M*. *cordata* as feed additives for improving the growth performance, survival rate, immune responses, and resistance to vibriosis of Pacific white shrimp.

## Introduction

Natural products from plants are becoming increasingly popularly utilized as feed additives in animal production for both terrestrial and aquatic species. Medicinal plants usually possess multiple biological activities such as antimicrobial, anti-inflammatory, antioxidant, immunostimulatory, and appetite-stimulating effects, which could potentially enhance the growth performance and health condition of animals [1–3]. Plant-derived isoquinoline alkaloids (IQs) are natural active components of various plants including *Macleaya cordata* or pink plume poppy, a herbaceous perennial plant in the family Papaveraceae which is widely distributed in China. *M*. *cordata* has long been used in traditional Chinese medicine as a topical agent for the treatment of inflammation and certain skin diseases [4–8].

The main bioactive isoquinoline alkaloids, namely sanguinarine and chelerythrine, are well known for their anti-inflammatory and antimicrobial properties [8,9]. It has generally been found that sanguinarine tends to be more active than chelerythrine with respect to these anti-inflammatory [10,11] and antimicrobial activities [7,12], although this result is not without exception [13]. Regarding the distribution of sanguinarine and chelerythrine within the plant, both alkaloids are found most abundantly in the capsules (32.08 and 7.36 mg/g dry weight, respectively) followed by the aerial part (4.51 and 2.88 mg/g dry weight, respectively), and at very low concentrations in the seeds (0.07 and 0.02 mg/g dry weight, respectively) [7]. Currently, IQs are considered promising feed additives to improve overall health condition and replace unnecessary antibiotic use in animal farming.

The potential health-promoting properties of IQ from *M*. *cordata* have been demonstrated in many animal species, including pigs [14], chickens [15–18], and fish [19–22]. These effects include growth performance enhancement, increased appetite, immunostimulation, alteration of the gut microbiome, and increased resistance to bacterial infection. For example, red tilapia fed with the IQ extracts at the rates of 25–100 mg/kg feed for 60 days had significantly higher final body weight (73.6–78.7 g), mean daily feed intake (1.19–1.25 g/fish/day), and leukocyte count (3.35–4.45 x 10^4^ cell/μL) than the control fish which were 57.8 g, 1.03 g/fish/day, and 1.61 x 10^4^ cell/μL, respectively [19]. Similarly, Caspian roach fed with the IQ extracts at the rates of 0.5–1.5 g/kg feed for 45 days had significantly higher final body weight (4.3–4.8 g) compared to the control (3.5 g) [20]. In addition, the IQ extracts (50–450 mg/kg feed) also showed anti-inflammatory and anti-oxidative effects, improved non-specific immunity, and enhanced resistance against *Aeromonas hydrophila* infection in koi carp [22]. Regarding the application of IQs in shrimp aquaculture, our previous study revealed that the IQ-fed Pacific white shrimp (100–200 mg/kg feed for 60 days and challenged with *Vibrio harveyi*) had a lower intestinal *Vibrio* spp. count compared to control shrimp, even though the effects on growth and survival were not significant [23]. We suspected that the limited effectiveness of the IQ extracts in promoting the growth and survival rate of the shrimp in our previous study might be related to the methodology of experimental diet preparation. Whereas our previous work utilized a simple top-coating approach which might result in the leaching of some IQ content from the pellet feed, the experimental feeds in the current study were made by mixing the IQ extracts with the other ingredients before pelleting to minimize the leaching problem of the IQs. Therefore, we expected that the health-promoting activity of the IQ extracts in the Pacific white shrimp in the present study would be more prominent.

The current study aimed to investigate under laboratory conditions the effects of two different formulations of IQ (a powdered formula (IQ-E; Sangrovit^®^ Extra) and a water-soluble, granulated formula (IQ-WS; Sangrovit^®^ WS), which contained the IQ sanguinarine as the main active compound at the concentrations of 0.5% and 1%, respectively) on the growth performance, survival rate, immune response, and resistance to *Vibrio parahaemolyticus* infection of Pacific white shrimp (*Litopenaeus vannamei*). The study was divided into two experiments. In Experiment 1, the effects of IQs on growth, survival, and the immune system were investigated in healthy shrimp. In Experiment 2, the effects of IQs on shrimp growth, survival, and resistance to *V*. *parahaemolyticus* infection were evaluated after challenge with *V*. *parahaemolyticus* via immersion. The result of the present study provides useful information regarding the effects of IQ extracts as a shrimp feed additive for sustainable shrimp culture.

## Materials and methods

### Experiment 1: Effects of IQ on growth performance, survival, and immune response of healthy shrimp

#### Preparation of the experimental diets

IQs were obtained from cleaned, sieved and ground *M*. *cordata* plant material by solvent extraction with ethanol. Following extraction, the liquid extract was filtered and gently dried, milled and mixed with carrier materials. Five experimental diets were formulated: commercial pelleted feed without IQ supplementation (control diet), feed supplemented with a preparation of powdered IQ (IQ-E; Sangrovit^®^ Extra) at 200 or 300 mg/kg of feed (providing 1 or 1.5 mg sanguinarine per kg of feed, respectively), and feed supplemented with a water-soluble, granulated preparation of IQ (IQ-WS; Sangrovit^®^ WS) at 100 or 150 mg/kg of feed (providing 1 or 1.5 mg sanguinarine per kg of feed, respectively). Each experimental diet was prepared by mixing either of the two IQ preparations with a small amount of distilled water and mixed with the other ingredients by a pelleting machine to make the IQ-containing pellet feed. The ingredients and proximate composition of the basal diet was shown in Table 1.

**Table 1:** Ingredients and proximate composition (%) of the basal diet used in the current study.

Ingredients	Percent
Fish meal	15.00
Soybean meal	42.15
Wheat bran	1.25
Corn protein concentrate	2.90
Wheat flour	23.30
Premix*	7.05
Binder	0.10
Squid liver powder	2.50
Fish soluble extract	1.50
Lecithin	2.75
Fish hydrolysate	1.50
**Proximate composition**	
Ash	15.44
Carbohydrate	28.36
Lipid	8.00
Moisture	10.12
Protein	38.08

Note

*Premix composition (per kg) as follows: Vitamin A 6,700,000 IU, vitamin D 1,350,000 IU, vitamin E 67 g, vitamin K3 3.4 g, vitamin B1 6.7 g, vitamin B2 10 g, vitamin B6 8 g, vitamin B12 13.5 g, niacin 53 g, pantothenic 26.5 g, folic acid 3.3 g, and biotin 335 g.

#### Experimental animals

A total of 3000 healthy Pacific white shrimp (*Litopenaeus vannamei*) postlarvae 9 (PL-9) were obtained from a commercial shrimp hatchery in Chachoengsao Province, Thailand, and were transported to the Aquaculture Business Research Center (ABRC) laboratory at the Faculty of Fisheries, Kasetsart University, Thailand. Following 3 days of acclimation at 29°C in a 500 L fiberglass tank with 25 ppt salinity, a total of 2000 PL-12 shrimp (about 1.5 mg/PL) were randomly distributed into 20 fiberglass tanks (five groups with four replicates/treatment group and 100 shrimp/tank), resulting in approximately 150 shrimp/m^2^. Throughout the study period, the water temperature was maintained at 26–27°C using an aquarium heater, with a salinity of 25 ppt, pH of 7–8, dissolved oxygen above 6 mg/L, alkalinity above 120 mg/L as CaCO_3_, total ammonia below 0.3 mg/L, and nitrite below 0.2 mg/L. Water quality parameters were analyzed weekly. Temperature and dissolved oxygen were measured by YSI PRO 20 (YSI Inc./Xylem Inc., Yellow Springs, OH, USA). Salinity was measured by a salinometer. pH was measured by EcoScan pH 5 (Thermo Fisher Scientific Inc.). Alkalinity, total ammonia, and nitrite were analyzed according to American Public Health Association, American Water Works Association, and Water Environment Federation [24]. All water parameters were suitable for Pacific shrimp culture and were presented in Table 2.

**Table 2:** Water quality parameters throughout the 30 day-feeding trial.

Water quality parameters	Treatment groups				
	Control	IQ-E 200 mg/kg feed	IQ-E 300 mg/kg feed	IQ-WS 100 mg/kg feed	IQ-WS 150 mg/kg feed
Temperature (°C)	26.80 ± 0.17^a^	26.69 ± 0.05^a^	26.76 ± 0.06^a^	26.78 ± 0.10^a^	26.78 ± 0.06^a^
Dissolved oxygen (mg/L)	6.64 ± 0.01^a^	6.66 ± 0.18^a^	6.72 ± 0.13^a^	6.48 ± 0.09^a^	6.57 ± 0.22^a^
pH	7.65 ± 0.22^a^	7.61 ± 0.18^a^	7.64 ± 0.13^a^	7.64 ± 0.09^a^	7.62 ± 0.22^a^
Alkalinity (mg/L as CaCO_3_)	143.75 ± 4.86^a^	144.50 ± 6.76^a^	149.25 ± 4.57^a^	147.50 ± 5.00^a^	147.50 ± 8.66^a^
Total ammonia (mg/L)	0.25 ± 0.11^a^	0.25 ± 0.07^a^	0.23 ± 0.02^a^	0.23 ± 0.11^a^	0.16 ± 0.05^a^
Nitrite (mg/L)	0.13 ± 0.01^a^	0.14 ± 0.01^a^	0.14 ± 0.01^a^	0.12 ± 0.01^a^	0.13 ± 0.01^a^

The data was presented as mean ± SD. Means with different superscripts in a row are significantly different from each other (p < 0.05).

#### Growth and survival study

The shrimp in each tank were fed *ad libitum* with one of the five experimental diets four times per day (at 8:00 a.m., 11:00 a.m., 2:00 p.m., and 5:00 p.m.) for 30 days. On days 10, 20, and 30 of the feeding trial, 10 shrimp per tank were randomly selected and weighed. The shrimps were weighted individually using a balance with 2 decimal places. The survival rate in each tank was also determined at the same time.

#### Immunological study

On day 30, five shrimp from each group were randomly selected for the immunological study. A blood sample of 0.2 mL was withdrawn from the base of the third walking leg of each sample shrimp using a syringe containing 0.6 mL of anticoagulant (K-199 + 5% l-cysteine). The immune parameters evaluated in the current study were total hemocyte count, phagocytic activity, phenoloxidase activity, and superoxide dismutase (SOD) activity.

Total hemocyte count was determined from the blood (100 μL) using a hemocytometer under a light microscope and calculated as total hemocytes/mL of hemolymph.

Phagocytic activity was determined following the procedure reported by Itami *et al*. [25]. The collected shrimp hemocytes were rinsed with shrimp saline, and the viable cell number was adjusted to 1 × 10^6^ cells/mL. The cell suspension (200 μL) was inoculated into a cover slip. After 20 min, the cell suspension was removed and rinsed three times with shrimp saline. Heat-killed yeast was added before incubation for 2 h. After incubation, the heat-killed yeast was removed, rinsed with shrimp saline five times to attain a concentration of 5 × 10^8^ cells/mL, and fixed with 100% methanol. The cover slip was then stained with Giemsa stain and mounted with Permount Mounting Medium. A total of 200 hemocytes were counted. Phagocytic activity was expressed as the percentage of phagocytic hemocytes among the total hemocytes.

The method of phenoloxidase activity determination was modified from the procedure reported by Supamattaya *et al*. [26]. The hemolymph-anticoagulant mixture (200 μL) was washed three times with shrimp saline and centrifuged at 1000 rpm, 4°C, for 10 min. The hemocyte lysate (HLS) was prepared from hemocytes in a cacodylate buffer, pH 7.4, using a sonicator at 30 amplitude for 5 s. The suspension was then centrifuged at 10,000 rpm, 4°C for 20 min, and the supernatant was collected. Subsequently, 200 μL of 0.1% trypsin in cacodylate buffer was mixed with 200 mL of HLS, followed by the addition of 200 μL of 4 mg/mL l-dihydroxyphenylalanine (l-DOPA) as the substrate. Enzyme activity was measured as the absorbance of dopachrome at 490 nm. The protein content in the HLS was measured following the method of Lowry *et al*. [27]. The phenoloxidase activity was calculated as the increase in optimum density/min/mg of protein.

Superoxide dismutase (SOD) activity of the blood (20 μL) was measured using an SOD Assay Kit (Sigma-Aldrich) according to the manufacturer’s instructions.

### Experiment 2: Effects of IQs on growth performance, survival, and resistance to *V*. *parahaemolyticus* infection of shrimp after immersion challenge

#### Experimental animals

After the completion of Experiment 1, the surviving shrimp (about 1.3 g) in the four IQ groups were transferred into new 16 fiberglass tanks (four replicates/treatment group and 30 shrimp/tank). Shrimp from the control group in Experiment 1 were randomly divided into two new groups: positive control (challenged with *V*. *parahaemolyticus*) and negative control (without *V*. *parahaemolyticus* inoculation). Thus, a total of 24 tanks (six groups with four replicates/treatment group) were used in Experiment 2. The animal husbandry and water quality parameters of Experiment 2 were similar to those of Experiment 1 throughout the study period.

#### Immersion Challenge with *Vibrio parahaemolyticus*

*Vibrio parahaemolyticus* (TISTR 1596) used in the current study was isolated from a diseased shrimp with vibriosis from a commercial farm in eastern Thailand. The bacteria were reisolated on tryptic soy agar (TSA) supplemented with 1.5% NaCl and incubated for 24 h at 35°C. The bacterial colonies were transferred into tryptic soy broth (TSB) supplemented with 1.5% NaCl and incubated for another 24 h at 35°C. The bacteria were then centrifuged at 1000 rpm for 15 min. The supernatant was discarded, and the bacterial pellet was resuspended in 1.5% NaCl. The bacterial suspension turbidity was adjusted to an optical density value of 0.02 at 540 nm (A_540_), equal to 10^5^ CFU/mL. It was then inoculated into the water of all tanks (except for the negative control group) to produce a final concentration of 10^3^ CFU/mL.

#### Growth and survival study

Three hours after the bacterial challenge, the shrimp in each tank were fed *ad libitum* with one of the five experimental diets four times/day (at 8:00 a.m., 11:00 a.m., 2:00 p.m., and 5:00 p.m.) as in Experiment 1 for 14 days. The shrimp body weight and the survival rates were determined at the end of the experiment.

#### Histopathological study

At the end of the experiment, five shrimp in each group were randomly selected and fixed in Davidson’s fixative for 48 h, and then transferred to 70% ethanol until they were processed for the histopathological study according to Bell and Lightner [28].

#### Statistical analysis

The data were analyzed by one-way analysis of variance (ANOVA) followed by Duncan’s multiple range test using IBM SPSS Statistics version 27 software (IBM Corporation, Armonk, NY, USA). Differences were considered statistically significant if *p* < 0.05.

## Results

### Experiment 1: Effects of IQs on growth performance, survival, and immune response of healthy shrimp

After feeding on the experimental diets for 30 days, the average shrimp body weight across all IQ-fed groups (i.e., IQ-E at 200 and 300 mg/kg of feed, and IQ-WS at 100 and 150 mg/kg of feed) and the control group was about 1.3 g (Table 3). No significant difference in shrimp body weight was observed between the five groups (*p* > 0.05). However, the average survival rates of all IQ-fed groups (ranging from 90.25–94.50%) were significantly greater (*p* < 0.05) than those of the control shrimp (83.00%) (Table 4).

**Table 3:** Effects of IQ extracts on growth performance of the healthy shrimp.

Treatment group	Body weight (g)		
	Day 10	Day 20	Day 30
**Control**	0.32 ± 0.01^a^	0.75 ± 0.04^a^	1.33 ± 0.02^a^
**IQ-E 200 mg/kg feed**	0.34 ± 0.01^a^	0.88 ± 0.02^a^	1.35 ± 0.01^a^
**IQ-E 300 mg/kg feed**	0.34 ± 0.02^a^	0.86 ± 0.01^a^	1.36 ± 0.02^a^
**IQ-WS 100 mg/kg feed**	0.33 ± 0.01^a^	0.84 ± 0.09^a^	1.34 ± 0.02^a^
**IQ-WS 150 mg/kg feed**	0.33 ± 0.03^a^	0.88 ± 0.03^a^	1.35 ± 0.03^a^

The data was presented as mean ± SD. Means with different superscripts in a column are significantly different from each other (p < 0.05).

**Table 4:** Effects of IQ extracts on survival rate of the healthy shrimp.

Treatment group	Survival rate (%)		
	Day 10	Day 20	Day 30
**Control**	100.00 ± 0.00^a^	94.50 ± 1.29^a^	83.00 ± 2.94^a^
**IQ-E 200 mg/kg feed**	100.00 ± 0.00^a^	98.00 ± 1.50^a^	94.50 ± 3.32^b^
**IQ-E 300 mg/kg feed**	100.00 ± 0.00^a^	95.00 ± 1.71^a^	93.25 ± 2.06^b^
**IQ-WS 100 mg/kg feed**	100.00 ± 0.00^a^	97.00 ± 2.38^a^	91.00 ± 2.16^b^
**IQ-WS 150 mg/kg feed**	100.00 ± 0.00^a^	95.00 ± 3.27^a^	90.25 ± 1.89^b^

The data was presented as mean ± SD. Means with different superscripts in a column are significantly different from each other (p < 0.05).

**Fig 1:**
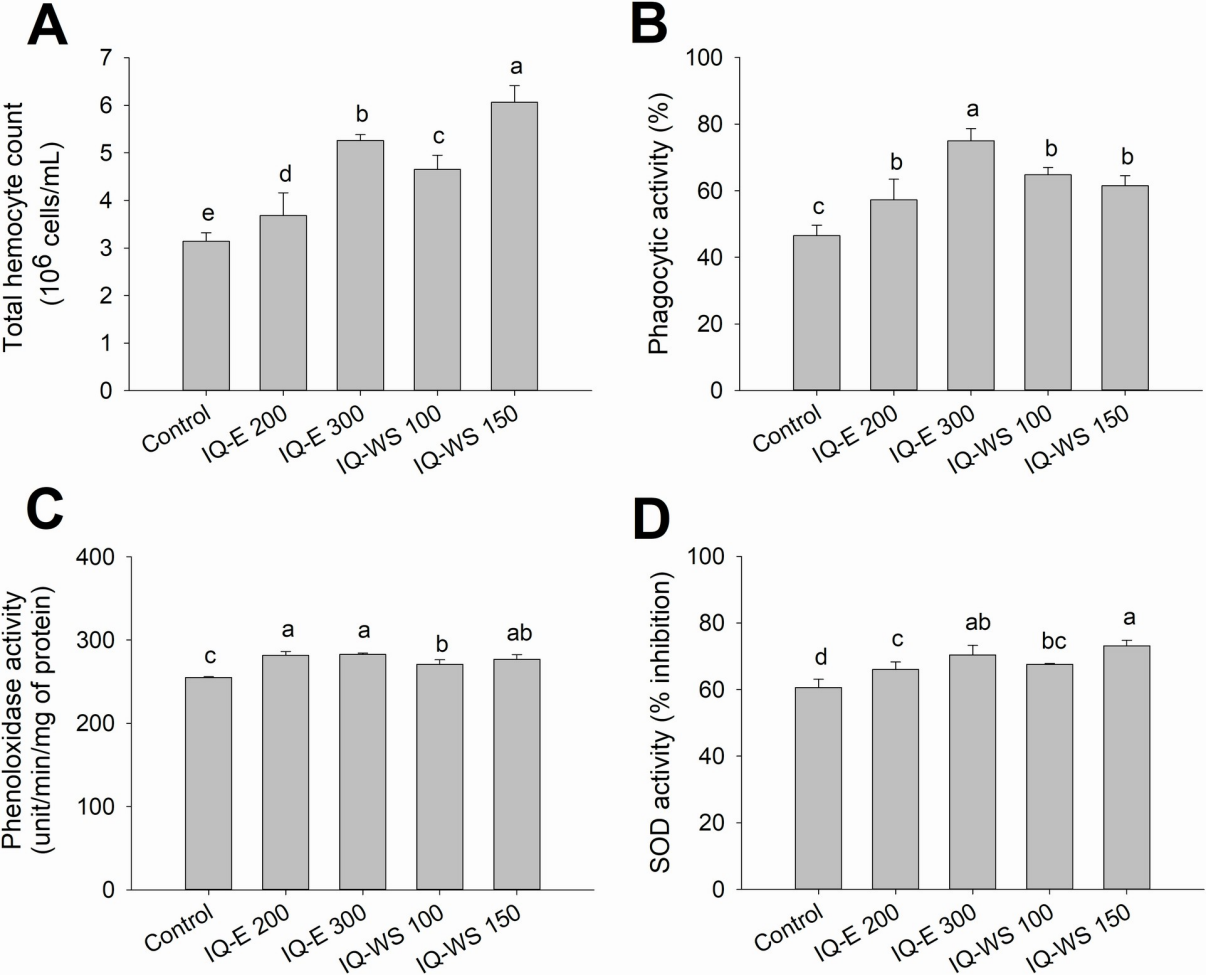
Effects of IQ extracts on immune parameters of the healthy shrimp. Total hemocyte count (10^6^ cells/mL) (**A**), phagocytic activity (%) (**B**), phenoloxidase activity (unit/min/mg of protein) (**C**), and superoxide dismutase (SOD) activity (% inhibition) (**D**) of the shrimp (*n* = 5) fed the control diet, IQ-E at 200 or 300 mg/kg of feed, and IQ-WS at 100 or 150 mg/kg of feed on day 30. The data are presented as the mean ± standard deviation. Different letters above the bars indicate significant differences (*p* < 0.05).

At the end of Experiment 1, all the IQ-supplemented groups showed a significant improvement (*p* < 0.05) in every immune parameter compared to the control shrimp. The total hemocyte counts of the control, IQ-E 200 and 300 mg/kg, and IQ-WS 100 and 150 mg/kg groups were 3.14, 3.68, 5.26, 4.56, and 4.65 × 10^6^ cells/mL, respectively (Fig 1A). The phagocytic activities were 46.50%, 57.25%, 75.00%, 64.75%, and 61.50%, respectively (Fig 1B). The phenoloxidase activities were 255.09, 281.55, 282.59, 270.74, and 276.82 units/min/mg of protein, respectively (Fig 1C). The superoxide dismutase (SOD) activities were 60.62%, 66.01%, 70.40%, 67.52%, and 73.12%, respectively (Fig 1D).

### Experiment 2: Effects of IQs on growth performance, survival, and resistance to *V*. *parahaemolyticus* infection of shrimp after immersion challenge

Following a further 14 days of the same experimental diets with the addition of *V*. *parahaemolyticus* immersion challenge at a concentration of 10^3^ CFU/mL, the average body weights of the IQ-E 200 and 300 mg/kg and IQ-WS 100 and 150 mg/kg groups were 1.59, 1.71, 1.61, and 1.67 g (Table 5), respectively, which were significantly higher (*p* < 0.05) than both the negative control (without *V*. *parahaemolyticus*) (1.39 g) and the positive control shrimp (with *V*. *parahaemolyticus*) (1.35 g). The survival rates of the IQ-fed groups were 87.50%, 90.00%, 85.00%, and 87.50% (Table 5), which were similar to the negative control shrimp (91.67%) but significantly higher than the positive control (68.33%) (*p* < 0.05). No significant difference was observed between the four IQ groups and the negative control (*p* > 0.05).

**Table 5:** Effects of IQ extracts on growth performance and survival rate of the *Vibrio parahaemolyticus*-infected shrimp.

Treatment group	Body weight (g)	Survival rate (%)
**Negative control (without *V*. *parahaemolyticus*)**	1.39 ±0 .09^b^	91.67 ± 1.92^a^
**Positive control (with *V*. *parahaemolyticus*)**	1.35 ±. 017^b^	68.33 ± 1.92^b^
**IQ-E 200 mg/kg feed**	1.59 ± 0.10^a^	87.50 ± 3.19^a^
**IQ-E 300 mg/kg feed**	1.71 ± 0.12^a^	90.00 ± 2.72^a^
**IQ-WS 100 mg/kg feed**	1.61 ± 0.04^a^	85.00 ± 3.33^a^
**IQ-WS 150 mg/kg feed**	1.67 ± 0.10^a^	87.50 ± 3.19^a^

The data was presented as mean ± SD. Means with different superscripts in a column are significantly different from each other (p < 0.05).

**Fig 2:**
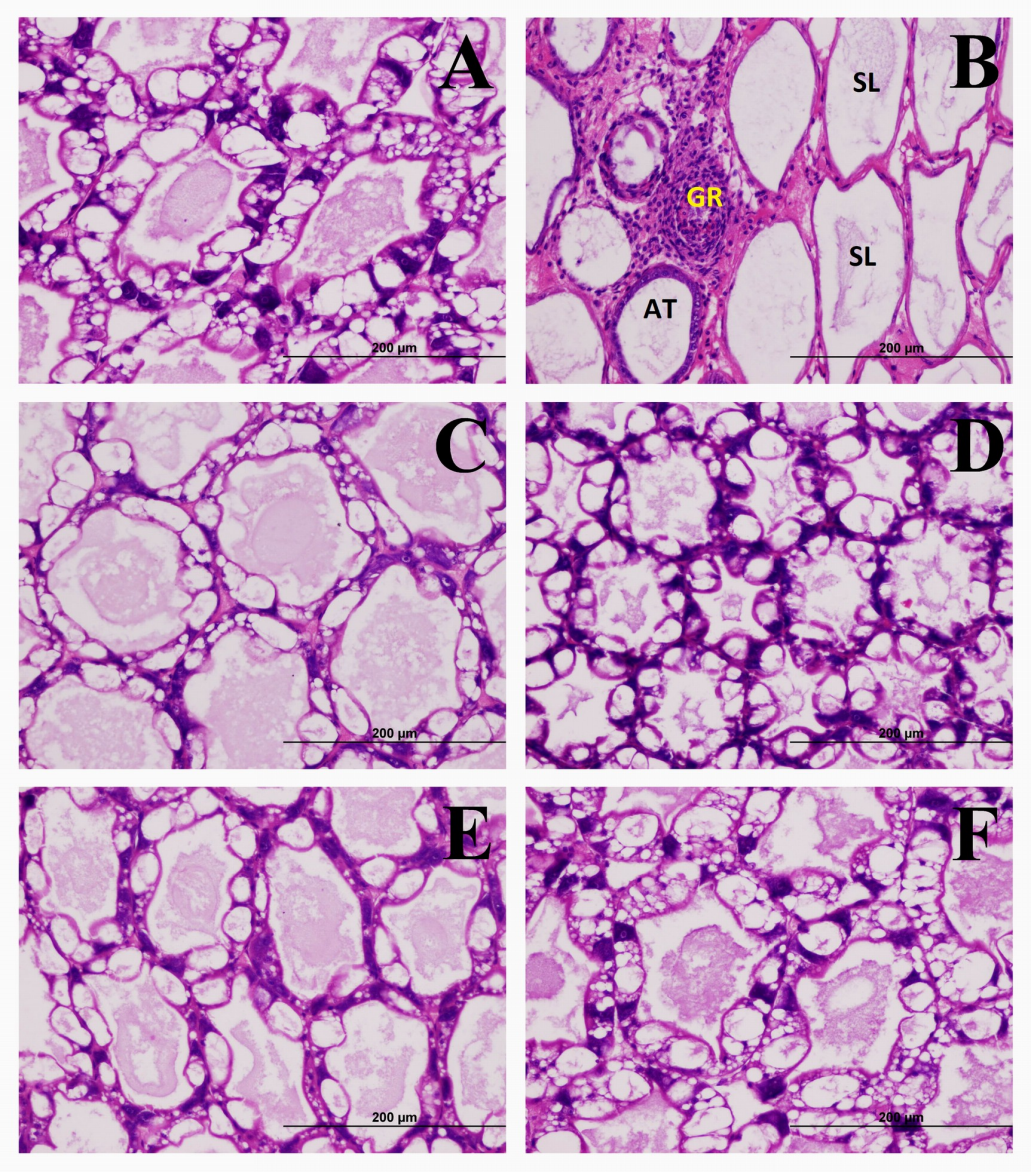
Effects of IQ extracts on histopathology of the hepatopancreas of the *Vibrio parahaemolyticus*-infected shrimp. Hepatopancreas of the shrimp fed the negative control diet (**A**), positive control diet showing atrophy (AT), sloughing of the hepatopancreas tubule epithelial cells (SL), and granulomatous encapsulation (GR) (**B**), IQ-E 200 mg/kg of feed diet (**C**), IQ-E 300 mg/kg of feed diet (**D**), IQ-WS 100 mg/kg of feed diet (**E**), and IQ-WS 150 mg/kg of feed diet (**F**) on day 14 after immersion challenge with *Vibrio parahaemolyticus* (10^3^ CFU/mL) (H&E stain).

**Fig 3:**
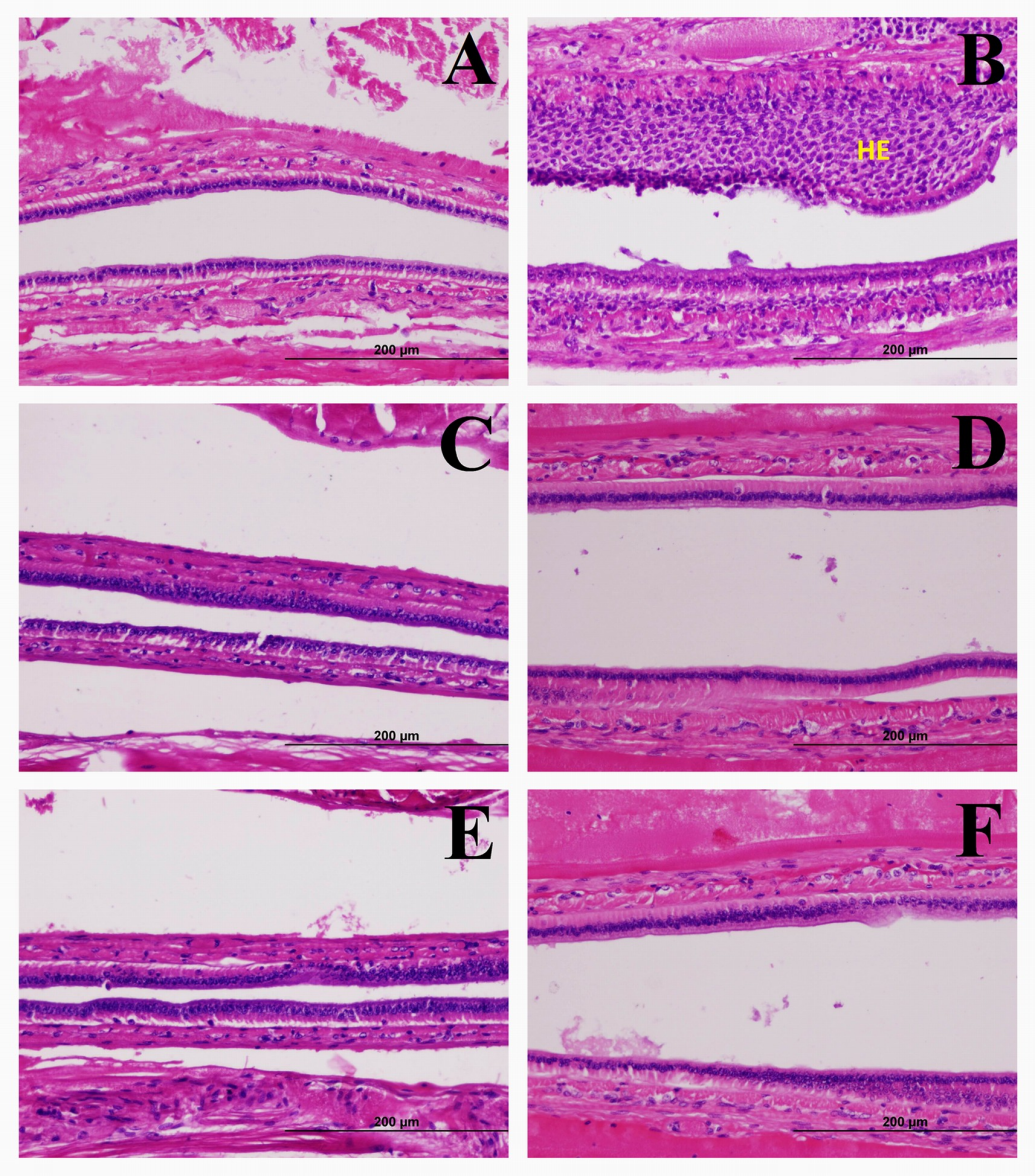
Effects of IQ extracts on histopathology of the intestine of the *Vibrio parahaemolyticus*-infected shrimp. Intestine of the shrimp fed the negative control diet (**A**), positive control diet showing hemocytic infiltration (HE) (**B**), IQ-E 200 mg/kg of feed diet (**C**), IQ-E 300 mg/kg of feed diet (**D**), IQ-WS 100 mg/kg of feed diet (**E**), and IQ-WS 150 mg/kg of feed diet (**F**) on day 14 after immersion challenge with *Vibrio parahaemolyticus* (10^3^ CFU/mL) (H&E stain).

The hepatopancreas and intestine tissues of the negative control shrimp and all IQ groups had a normal appearance and no signs of bacterial infection (Figs 2 and 3). In contrast, the hepatopancreas tissues of the positive control shrimp showed signs of bacterial infection such as atrophy, sloughing of the hepatopancreas tubule epithelial cells, and granulomatous encapsulation. Unsurprisingly, the intestinal tissues of the positive control shrimp, but not of the other groups, showed signs of hemocytic infiltration in the submucosa due to bacterial infection. The finding that no histopathological change was observed in all IQ-fed groups was consistent with the higher survival rates of these groups. Taken together, they indicated better resistance to *V*. *parahaemolyticus* infection as a result of IQ extracts.

## Discussion

The mechanisms of phytochemicals in improving overall animal health have yet to be firmly established, as they most likely involve many complex interactions between the plant compounds and the host factors. Nevertheless, it is arguable that plant secondary metabolites may exert health-improving effects in a similar fashion to antibiotic growth promoters (AGPs), to a certain degree. Currently, the main hypothesis for the growth-promoting effect of AGPs is the antimicrobial hypothesis, which postulates that the effect may be related to the inhibition of subclinical infection even though the concentration of active compounds may be lower than the minimum inhibitory concentration (MIC) of the bacteria [29–31]. In fact, drug concentrations at the sub-MIC level can still affect bacterial growth profile, virulence factor expression, and susceptibility to the host immune system [31]. Possibly the strongest evidence in support of the antimicrobial hypothesis is that AGPs cannot promote the growth of germ-free animals [32]. An alternative explanation is the anti-inflammatory hypothesis, which attributes the growth-promoting effect to the suppression of intestinal inflammation. Niewold [33] argued that the two commonly used groups of AGPs, namely tetracyclines and macrolides, also have significant anti-inflammatory properties. By suppressing the production of proinflammatory cytokines and tissue catabolism, more energy is conserved and available for growth. Given that these two hypotheses are not mutually exclusive, it could be speculated that both antimicrobial and anti-inflammatory mechanisms may be involved in the growth-enhancing properties of AGPs and phytochemicals alike.

The IQ formulations tested in the present study contained 0.5% and 1% sanguinarine, respectively. When incorporated into the pellet feeds, the shrimp diets contained sanguinarine at doses of 1 or 1.5 mg/kg of feed (see Materials and Methods). Since sanguinarine and other alkaloids found in *M*. *cordata* have antimicrobial and anti-inflammatory activities [8], it can potentially be used as a growth promoter. In fact, many previous studies have demonstrated the growth-stimulating effect of IQs in a variety of animal species including pigs [14], chickens [15–18], and fishes [19–21]. In line with these studies, our results revealed growth-enhancing effects of the four IQ-supplemented diets. It is worth mentioning that only the *V*. *parahaemolyticus*-infected shrimp fed IQs (in Experiment 2) showed a significant improvement in body weight compared to the control group, whereas the healthy shrimp (in Experiment 1) did not. These observations support the antimicrobial and anti-inflammatory hypotheses mentioned above as the main mechanisms for the growth-promoting effect of IQs [29–31].

In contrast to the present study, our previous research did not show the improved growth performance and survival rates of shrimp that were administered IQ-WS at 100 and 200 mg/kg of feed diets [23]. A possible explanation for the previous ineffective outcomes may be related to the different methods of experimental feed preparation. While the preparations were mixed with the feed ingredients prior to pelleting in the present study, the previous experiment employed a top-coating method without adding an oil binder which might result in significant leaching of IQs from the prepared diets, thereby rendering the administered doses of the IQ lower than expected. Therefore, to prevent leaching and obtain the best result, IQ preparations should be incorporated into shrimp feed mash before making the pellet feed instead of utilizing a simple top-dressing method.

Herbal extracts have long been studied as enhancers of the immune functions of aquatic animals with promising results, even though the exact molecular mechanisms are often unknown. The effects of plant immunostimulants usually include stimulation of phagocytosis, as well as increased nonspecific immunity mediators [34–36]. Our result demonstrated the immunostimulatory effect of IQs on total hemocyte count as well as phagocytic, phenoloxidase, and SOD activities of the shrimp hemocytes. Similarly, IQ-supplemented diets have been reported to increase total leukocyte count in red tilapia [19] and common carp [21], increase SOD, lysozyme, and complement activities in koi carp [22], and enhance antibody production in chickens [17] and pigs [14]. These improved immune responses may have partly contributed to the higher survival rates of the IQ-fed shrimp in Experiments 1 and 2.

In addition to enhanced immune function, the higher survival rates of the shrimp could be attributed to the antimicrobial activity of the active compound sanguinarine. Sanguinarine has been found to affect the bacterial cell membrane [37] and interfere bacterial cytokinesis [38]. The MICs of sanguinarine have been reported in the range of 12.5–50 μg/mL against fish pathogenic bacteria (*Aeromonas hydrophila*, *A*. *salmonicida*, *Vibrio anguillarum*, and *V*. *harveyi*) [39], 15.6–62.5 μg/mL against human pathogenic bacteria (*Staphylococcus aureus*, *Escherichia coli*, and *Streptococcus agalactiae*) [39], 3–50 μg/mL against *Vibrio* spp. (*V*. *cholerae*, *V*. *parahaemolyticus*, and non-agglutinable *Vibrio* spp.) [40], and 6.25–50 μg/mL against *Helicobacter pylori* [12]. Intraperitoneal administration of sanguinarine to *A*. *hydrophila*-infected common carp at doses of 10–20 mg/kg body weight resulted in lower tissue bacterial loads and higher survival rates of the carp [41]. In addition to the antibacterial property, sanguinarine and *Macleaya* extracts also possesses anti-parasitic activity against fish ectoparasites such as *Ichthyophthirius multifiliis* [42,43], *Dactylogyrus intermedius* [44], and *Gyrodactylus kobayashii* [45].

Although the direct evidence of antimicrobial activity of sanguinarine or IQ extracts against shrimp pathogens has yet to be reported so far, it is most likely that sanguinarine or IQ extracts may also exert such effect. Our previous work revealed a significant reduction of the intestinal *Vibrio* spp. count of the *V*. *harveyi*-infected shrimp that were fed IQ-WS at 100 and 200 mg/kg of feed, although the survival rates were not significantly improved [23]. However, the shrimp that were fed IQ-supplemented diets in the present study showed better resistance to *V*. *parahaemolyticus* infection, as suggested by the higher survival rates of all IQ-fed groups compared to the *V*. *parahaemolyticus*-infected control shrimp. The most likely explanation for the inconsistency between the two studies (in terms of survival rates) is associated with the different methods of experimental feed preparation, as mentioned above. The absence of histopathological lesions in the hepatopancreas and intestinal tissues of the IQ-fed shrimp but not the positive control shrimp also supports with the protective effect of IQs.

The bioavailability of sanguinarine and other IQs from *M*. *cordata* in shrimp and other aquatic animals has yet to be studied. However, it was reported that they were very poorly absorbed from the gastrointestinal (GI) tract of the pig [46] and rat [47] following oral administration. This characteristic is desirable for the prevention of digestive tract infection, but may not suitable for systemic infection. Given that the GI tract is one of the major routes of *Vibrio* spp. infection in the shrimp after immersion challenge [48], it is reasonable to assume that the oral administration of IQ extracts could prevent *Vibrio* spp. colonization in the shrimp’s GI tract, thereby suppressing the disease outbreak which was the case in the present study.

## Conclusions

The two IQ formulations at the two sanguinarine concentrations (1 and 1.5 mg/kg of feed) supplemented into the shrimp diets showed equivalent efficacies in terms of improving growth performance, survival rate, immune response, and resistance to *V*. *parahaemolyticus* infection of Pacific white shrimp. Consequently, this natural product can be applied as a feed additive to support overall health condition for sustainable shrimp production.

## Supporting information

S1 Fig Effects of IQ extracts on growth performance and survival of the healthy shrimp. The body weight (g) (*n* = 10) (**A**) and survival rates (%) (*n* = 4) (**B**) of the shrimp fed the control diet, powdered isoquinoline alkaloids (IQ-E) at 200 or 300 mg/kg of feed, and water-soluble, granulated isoquinoline alkaloids (IQ-WS) at 100 or 150 mg/kg of feed on days 10, 20, and 30. The data are presented as the mean ± standard deviation. Different letters above the bars indicate significant differences (*p* < 0.05).
(TIF)

S2 Fig Effects of IQ extracts on growth performance and survival of the Vibrio parahaemolyticus-infected shrimp. The body weight (g) (*n* = 10) (**A**) and survival rates (%) (*n* = 4) (**B**) of the shrimp fed the control diet, IQ-E at 200 or 300 mg/kg of feed, and IQ-WS at 100 or 150 mg/kg of feed on day 14 after immersion challenge with *Vibrio parahaemolyticus* (10^3^ colony-forming units (CFU)/mL). The data are presented as the mean ± standard deviation. Different letters above the bars indicate significant differences each other (*p* < 0.05).
(TIF)
