# Supplementary figures and images for: Effects of isoquinoline alkaloids from Macleaya cordata on growth performance, survival, immune response, and resistance to Vibrio parahaemolyticus infection of Pacific white shrimp (Litopenaeus vannamei)
Source: PLoS One. 2021 May 6;16(5):e0251343. doi: 10.1371/journal.pone.0251343 (PMC8101937; doi:10.1371/journal.pone.0251343)

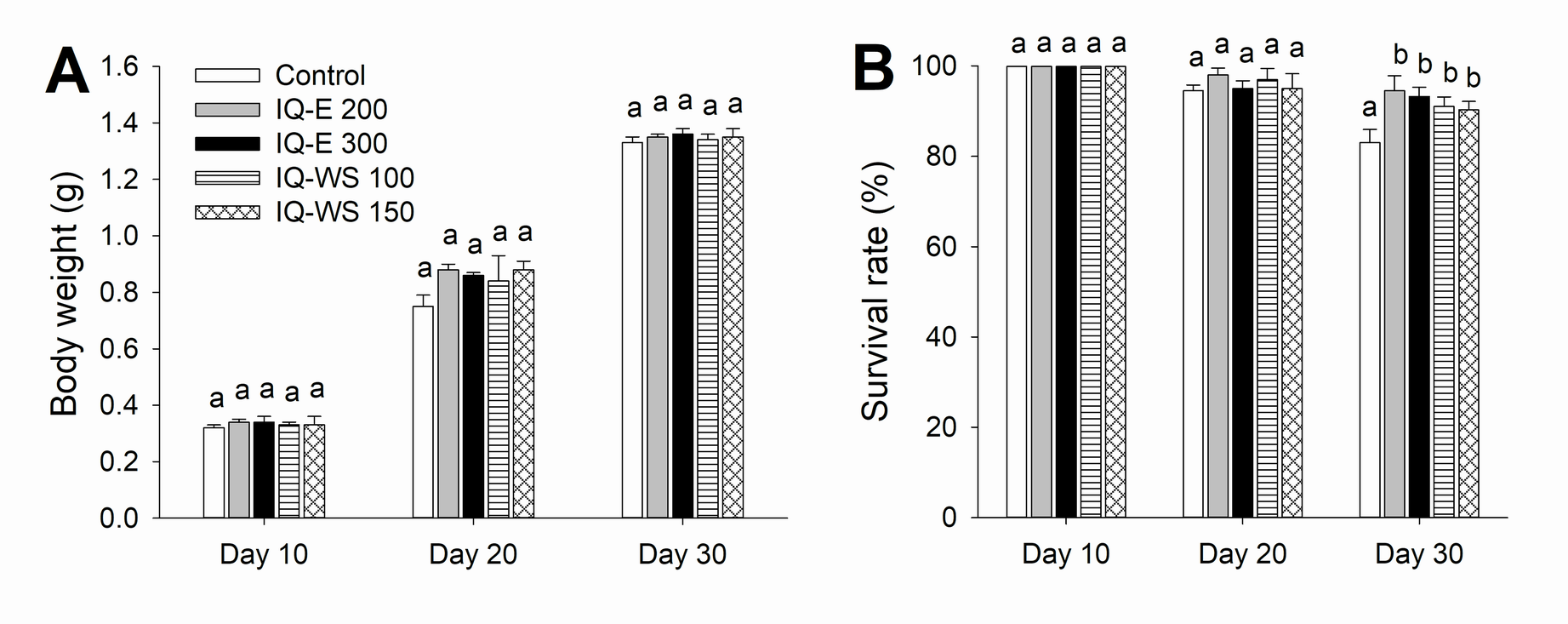

Supplement: S1 Fig — The body weight (g) (n = 10) (A) and survival rates (%) (n = 4) (B) of the shrimp fed the control diet, powdered isoquinoline alkaloids (IQ-E) at 200 or 300 mg/kg of feed, and water-soluble, granulated isoquinoline alkaloids (IQ-WS) at 100 or 150 mg/kg of feed on days 10, 20, and 30. The data are presented as the mean ± standard deviation. Different letters above the bars indicate significant differences (p < 0.05). (TIF) [file pone.0251343.s001.TIF]

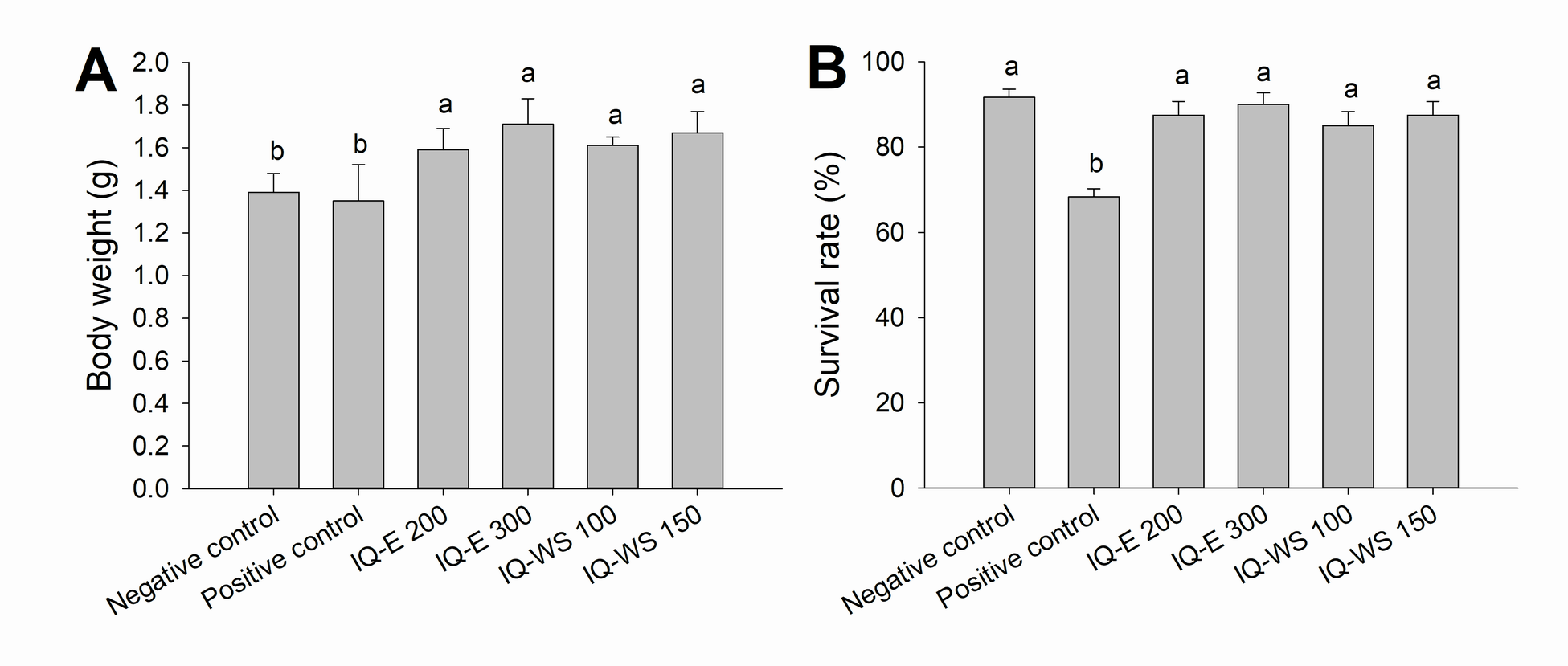

Supplement: S2 Fig — The body weight (g) (n = 10) (A) and survival rates (%) (n = 4) (B) of the shrimp fed the control diet, IQ-E at 200 or 300 mg/kg of feed, and IQ-WS at 100 or 150 mg/kg of feed on day 14 after immersion challenge with Vibrio parahaemolyticus (103 colony-forming units (CFU)/mL). The data are presented as the mean ± standard deviation. Different letters above the bars indicate significant differences each other (p < 0.05). (TIF) [file pone.0251343.s002.TIF]
